# Insecticide Resistance Monitoring in Field Populations of the Whitebacked Planthopper *Sogatella furcifera* (Horvath) in China, 2019–2020

**DOI:** 10.3390/insects12121078

**Published:** 2021-11-30

**Authors:** Zhao Li, Yao Qin, Ruoheng Jin, Yunhua Zhang, Zhijie Ren, Tingwei Cai, Chang Yu, Yu Liu, Yongfeng Cai, Qinghong Zeng, Hu Wan, Jianhong Li

**Affiliations:** Hubei Insect Resources Utilization and Sustainable Pest Management Key Laboratory, College of Plant Science and Technology, Huazhong Agricultural University, Wuhan 430070, China; Lucky_edmund@163.com (Z.L.); qinyao1246282025@163.com (Y.Q.); jinshirley@126.com (R.J.); zhangyunh6688@126.com (Y.Z.); rzj@webmail.hzau.edu.cn (Z.R.); caitingwei@webmail.hzau.edu.cn (T.C.); ycliule@163.com (C.Y.); 15071403650@163.com (Y.L.); yongfengcai1996@163.com (Y.C.); zqh854597499@163.com (Q.Z.); huwan@mail.hzau.edu.cn (H.W.)

**Keywords:** *Sogatella furcifera*, insecticide, insecticide resistance, detoxification enzymes, correlation analysis

## Abstract

**Simple Summary:**

The whitebacked planthopper (WBPH), *Sogatella furcifera* (Horváth), is one of the most destructive pests that seriously threatens the high-quality and safe production of rice. Overuse of chemical insecticides has led to varying levels of resistance to insecticides in the field population of *S. furcifera*. In this study, we measured the susceptibility of 18 populations to 10 insecticides by the rice-seedling dip method. Enzyme assays were performed to measure the levels of esterase (EST), glutathione *S*- transferase (GST) and cytochrome P450 monooxygenase (P450). A risk of cross-resistance between some insecticides were found by pairwise correlation, and EST may be contributed to the resistance to nitenpyram, thiamethoxam and clothianidin in *S. furcifera.* Overall, our findings will help inform the effective insecticide resistance management strategies to delay the development of insecticide resistance in *S. furcifera*.

**Abstract:**

Monitoring is an important component of insecticide resistance management. In this study, resistance monitoring was conducted on 18 field populations in China. The results showed that *S. furcifera* developed high levels of resistance to chlorpyrifos and buprofezin, and *S. furcifera* showed low to moderate levels of resistance to imidacloprid, thiamethoxam, dinotefuran, clothianidin, sulfoxaflor, isoprocarb and ethofenprox. *Sogatella furcifera* remained susceptible or low levels of resistance to nitenpyram. LC_50_ values of nitenpyram and dinotefuran, imidacloprid, thiamethoxam, clothianidin and chlorpyrifos exhibited significant correlations, as did those between dinotefuran and thiamethoxam, clothianidin, sulfoxaflor, imidacloprid, isoprocarb and buprofezin. Similarly, significant correlations were observed between thiamethoxam and clothianidin, sulfoxaflor and imidacloprid. In addition, the activity of EST in field populations of *S. furcifera* were significantly correlated with the LC_50_ values of nitenpyram, thiamethoxam and clothianidin. These results will help inform effective insecticide resistance management strategies to delay the development of insecticide resistance in *S. furcifera*.

## 1. Introduction

The whitebacked planthopper (WBPH), *Sogatella furcifera* (Horváth: *Delphacidae*, *Sogatella*), is one of the most destructive pests of rice crops and is widely distributed in Asian countries [1,2], causing serious damage to rice mainly by sucking at the base of rice stems and spreading viral diseases [3,4]. Chemical insecticides have a long history of controlling the WBPH, and they are still the primary measure of prevention and management [2,5,6]. However, due to long-term use of synthetic insecticides, various populations of WBPH have developed resistance to 15 compounds worldwide, including organophosphates, pyrethroids, carbamates, insect growth regulators and neonicotinoids [7,8,9]. Consequently, it is important to determine the resistance level of the field population of WBPH to frequently used insecticides, which are crucial for the successful management of the WBPH.

Previous studies have demonstrated an altered target site and enhanced detoxification (metabolism) as the main mechanisms of resistance to insecticides [10,11,12]. In *S. furcifera*, metabolic resistance due to overexpression of cytochrome P450 (monooxygenase) has contributed to neonicotinoid, insect growth regulator and organophosphate resistance, and an elevated level of GST has also been involved in resistance to neonicotinoids and insect growth regulator resistance [13,14]. In addition, increasing activities of EST have contributed to insect growth regulator resistance in *S. furcifera* [15].

The WBPH has become increasingly damaging in rice producing areas in the Yangtze River region, which is the main paddy field in China [8]. In our study, we assessed the resistance situation of *S. furcifera* to 10 insecticides (including carbamates, pyrethroids, neonicotinoids, organophosphates and insect growth regulators) from 18 areas of six provinces in China, and analyzed the potential cross-resistance patterns of these insecticides in all collected field populations of *S. furcifera*. We also determined the correlation between the activity of detoxification enzymes and the susceptibilities of *S. furcifera* field populations. The findings of this study not only provide directions for the sustainable control of this important pest in rice producing areas, but also provide useful data for the insecticide resistance management and integrated pest management (IPM).

## 2. Materials and Methods

### 2.1. Insect Populations

Eighteen field populations of WBPH were collected from rice paddy fields in six provinces in China (Table 1). Insects were reared under the following conditions: temperature of 27 ± 1 °C, relative humidity (RH) of 70–80% and photoperiod of 16:8 h light/dark. The field-collected WBPH was mass mated and maintained in the laboratory for 1–2 generations, and the progeny were used for the susceptibility bioassay and enzyme assays.

### 2.2. Insecticides

The 10 insecticides were sourced and tested: nitenpyram (Hubei Kangbaotai Fine-Chemicals Co., Ltd., Wuhan, China, 95.8%), dinotefuran (Hubei Kangbaotai Fine-Chemicals Co., Ltd., 91.0%), thiamethoxam (Hubei Kangbaotai Fine-Chemicals Co., Ltd., 95%), clothianidin (Hubei Kangbaotai Fine-Chemicals Co., Ltd., Wuhan, China, 96.0%), sulfoxaflor (Dow AgroSciences Inc., Shanghai, China, 97.9%), imidacloprid (Hubei Kangbaotai Fine-Chemicals Co., Ltd., Wuhan, China, 95.8%), chlorpyrifos (Hebei VeYong Bio-Chemical Co., Ltd., Shijiazhuang, China, 98.0%), isoprocarb (Jiangsu Changlong Chemicals Co., Ltd., Changzhou, China, 97.9%), ethofenprox (Suzhou ATL Chemical Co., Ltd., Suzhou, China, 95.0%) and buprofezin (Jiangsu Anpon Electrochemical Co., Ltd., Huai’an, China, 97.4%). The modes of action included nitenpyram, dinotefuran, thiamethoxam, clothianidin, sulfoxaflor and imidacloprid via nicotinic acetylcholine receptor (nAChR) competitive modulators; chlorpyrifos and isoprocarb via acetylcholinesterase (AChE) inhibitors; ethofenprox and buprofezin via sodium channel modulators and inhibitors of chitin biosynthesis, respectively [16].

### 2.3. Bioassays

Bioassays were performed with third-instar nymphs of *S. furcifera* using a rice-seedling dip method that has been described previously [17]. Briefly, fifteen rice seedlings were combined, immersed in a concentration of insecticide dilutions (Table S1) for 30 s and then air-dried at 25 °C for more than 30 min. The roots of rice seedlings were wrapped with cotton moistened with water, and placed in a 500-mL plastic cup. Each replicate contained fifteen third-instar nymphs. Three replicates of each concentration and six replicates of each insecticide were carried out, with rice seedlings dipped in water containing 0.1% Triton X-100 (Sigma–Aldrich, St. Louis, MO, USA). Triton X-100 was used as a control. Mortality was counted after exposure to isoprocarb, chlorpyrifos, and ethofenprox for 72 h, imidacloprid, thiamethoxam, dinotefuran, clothianidin, sulfoxaflor, and nitenpyram for 96 h, and buprofezin for 120 h.

### 2.4. Enzyme Assays

EST activity was determined using the method described previously [7]. Approximately 0.02 g of third-instar nymphs for each repetition was homogenized in 1 mL of ice-cold sodium phosphate buffer (0.04 M, pH 7.0) and centrifuged at 4 °C and 14,000 rpm for 10 min. The homogenized supernatant was then carefully transported to a new Eppendorf tube and used as the enzyme source. A total of 1.2 mL of substrate solution (containing 1 mL 3 × 10^−4^ M α-NA and 0.2 mL of the enzyme source) was added and then incubated at 37 °C for 15 min. The reaction was terminated by the addition of 0.2 mL of the dye reagent. An NP80 nanophotometer (IMPLEN, Munich, Germany) was used to measure the optical density at 600 nm.

GST activity was assayed as previously described [7]. The enzyme solution was prepared as it was in the EST assay. For each reaction, 740 µL of sodium phosphate buffer (0.1 M, pH 6.5), and 30 µL of 30 mM 1-chloro-2,4-dinitrobenzene (CDNB), 100 µL of enzyme solution, 30 µL of 30 mM GSH were mixed. The absorbance was recorded using an NP80 NanoPhotometer (IMPLEN, Munich, Germany) at 340 nm for 2 min.

The 7-ethoxycoumarin-O-deethylase (7-ECOD) activity of P450 was determined as described previously with slight modification [18]. Approximately 0.2 g of the third-instar nymphs was homogenized in 1.0 mL of 0.1 M sodium phosphate buffer containing 1 mM ethylenediaminetetraacetic acid EDTA (Sinopharm Chemical Reagent Co., Ltd., Shanghai, China) 1 mM phenylmethylsulfonyl fluoride (PMSF), 1 mM dithiothreitol (DTT) and 10% glycerol, and then centrifuged at 4 °C 14,000 rpm for 15 min. The supernatant was centrifuged at 4 °C 14,000 rpm for 30 min again. For the reaction, 125 µL of crude homogenate was mixed with 365 μL of 0.1 M sodium phosphate buffer (pH 7.5), 10 μL of 10 mM aqueous NADPH and 5 µL of 40 mM 7-ECOD and then incubated at 30 °C for 15 min. The reaction was terminated by adding 150 µL of 15% trichloroacetic acid (TCA). Then, the mixture was centrifuged, and 1.6 mM glycine-NaOH buffer (pH 10.5) was added. A Spark 10 M Multimode Microplate Reader (Tecan, Männedorf, Switzerland) was used for measuring the fluorescence intensity with an excitation wavelength of 358 nm and an emission wavelength of 465 nm. The protein content of the enzyme solutions was determined using Quick Start™ Bradford 1*Dye Reagent (Bio-Rad Laboratories, Inc., Hercules, CA, USA).

### 2.5. Data Analyses

Abbott’s formula was used to control mortality for the mortality data obtained [19]. The slopes with standard errors (SE), and LC_50_ values with 95% confidence intervals, were calculated by probit analysis. The LC_50_ of the most susceptible population was confirmed in previous studies in our lab [7]. The classification standard of resistance level was based on the “Technological rules for monitoring insecticide resistance in rice white-backed planthopper, *Sogatella furcifera*” (NY/T 3159-2017), Agricultural industry standard of the people’s Republic of China. The resistance ratio (RR): RR ≤ 5, 5 < RR ≤ 10, 10 < RR ≤ 100 and RR > 100 were classified as susceptible, low level of resistance, moderate level of resistance, and high level of resistance, respectively. The LC_50_ values of the susceptibility baseline and minimum recommended dose of registered insecticides to *S. furcifera* are listed in Table 2. Spearman correlations between the LC_50_ values of different insecticides were calculated using the WGCNA package (version 1.69) and visualized using corrplot (version 0.1.3). The relative enzyme activities and differences in mortality were analyzed using unpaired Student’s t-tests with at least three repeats. The standard of statistically significant differences was *p* < 0.05.

## 3. Results

### 3.1. Insecticide Resistance

**Figure 1:**
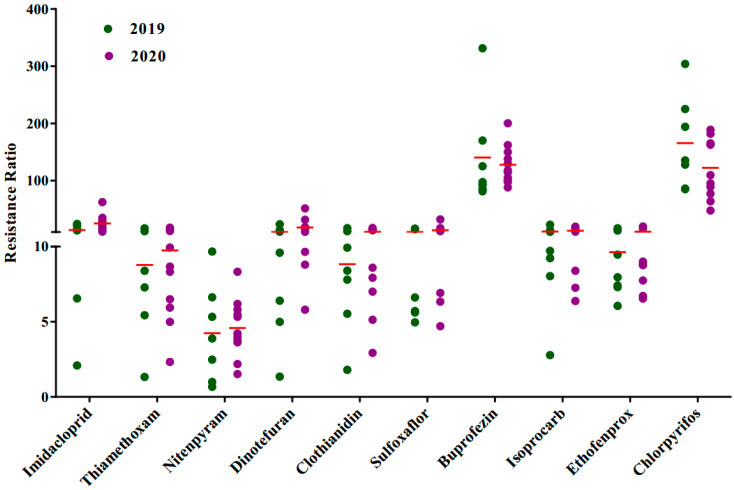
Resistance levels of field populations of *S. furcifera* to frequently used insecticides. Red horizontal lines across the scatter diagram represent the mean values of the resistance ratio of the different populations.

To determine the resistance level of *S. furcifera* in rice paddy fields in China, we measured the susceptibility of 18 populations to 10 insecticides by the rice-seedling dip method. The results indicated that *S. furcifera* had moderate to high levels of resistance to chlorpyrifos (RR = 47.75–304.17-fold) and buprofezin (RR = 81.25–331.50-fold). Furthermore, *S. furcifera* developed low to moderate resistance levels to dinotefuran (RR = 1.35–51.45-fold), thiamethoxam (RR = 1.33–16.61-fold), clothianidin (RR = 1.80–17.33-fold), sulfoxaflor (RR = 4.70–32.26-fold), imidacloprid (RR = 2.09–62.55-fold), isoprocarb (RR = 2.78–22.85-fold) and ethofenprox (RR = 6.06–19.62-fold). In addition, the resistance level of *S. furcifera* to nitenpyram (RR = 0.67–9.67-fold) remained susceptible to low levels (Table 3, Figure 1).

### 3.2. Enzyme Activity

**Figure 2:**
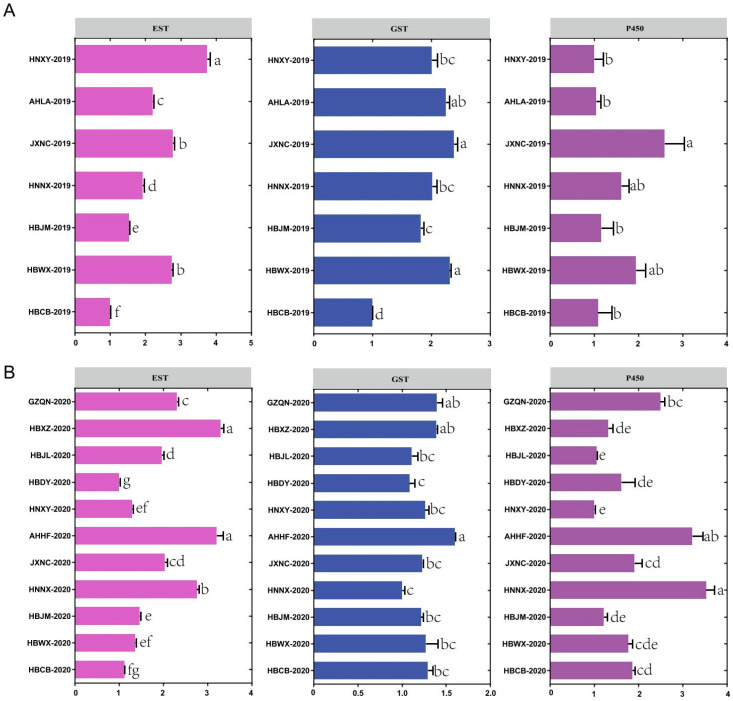
Relative activities of the detoxification enzymes in field populations of *S. furcifera* in 2019 (**A**) and 2020 (**B**). The relative enzyme activity of the detoxification enzyme corresponds to the ratio of the detoxification enzyme activity and the minimum enzyme activity. The SE bars with different small letters represent significant differences (*p* < 0.05).

To determine the role of detoxification enzymes in the insecticide resistance of *S. furcifera*, enzyme assays were performed to measure the levels of EST, GST and P450 (Figure 2A,B). The results showed that the EST activities from the populations of HNXY-2019 and HBCB-2019 exhibited a 3.75-fold difference in 2019, while the maximum difference in 2020 was between the HBXZ-2020 population and the HBDY-2020 population, and there was a 3.30-fold difference. The GST activities from the populations of JXNC-2019 and HBCB-2019 resulted in 2.38-fold variation, while the GST activities of the population from AHHF-2020 were 1.60-fold higher than those of the HNNX-2020 population. The P450 activities resulted in a 2.60-fold variation between the populations from JXNC-2019 and HNXY-2019 in 2019 and a 3.53-fold variation between the populations from HNNX-2020 and HNXY-2020 in 2020.

### 3.3. Pairwise Correlation Analysis

**Figure 3:**
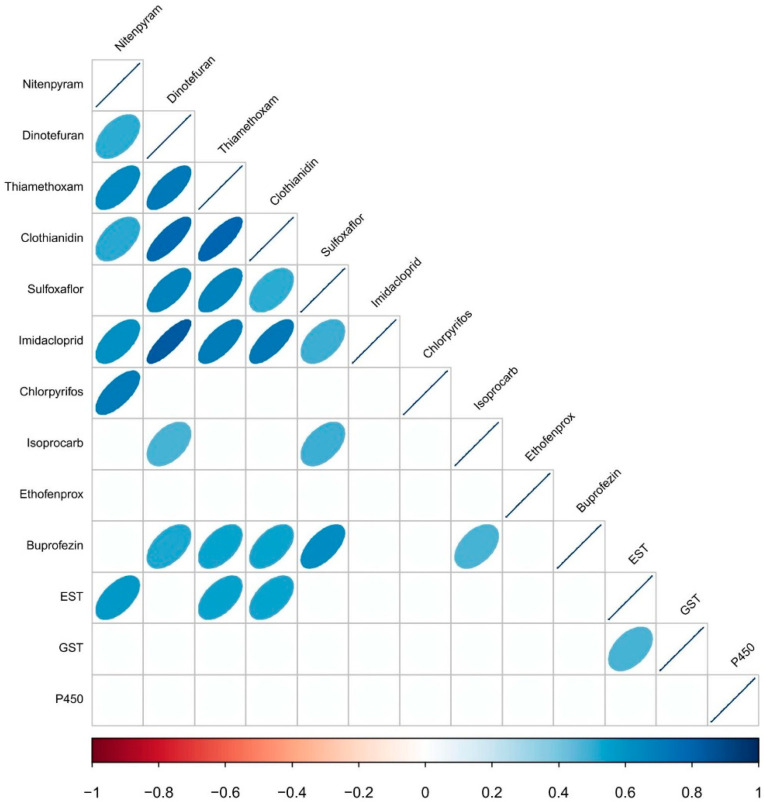
Correlations between the LC_50_ values of the tested insecticides and the enzyme activities of the field populations of *S. furcifera*. Correlations are based on linear Spearman correlation coefficients. The scale color of the filled squares indicates the strength of the correlation (r) and whether it is negative (red) or positive (blue). Only significant correlations with *p* < 0.05 are shown.

To determine whether there were similar insecticidal patterns between insecticides, the logarithmic values of the LC_50_ measurements of the two insecticides were compared by pairwise correlation coefficients (Figure 3). Resistance to imidacloprid was significantly correlated with neonicotinoid insecticides, such as dinotefuran, thiamethoxam, clothianidin, nitenpyram and sulfoximine insecticide sulfoxaflor. Similarly, there were significant positive correlations between resistance to the insect growth regulator insecticides buprofezin and dinotefuran, thiamethoxam, clothianidin, sulfoxaflor, and isoprocarb. Moreover, significant correlations were found between isoprocarb and dinotefuran, sulfoxaflor and buprofezin.

In addition, we analyzed the correlation between the activity of three primary detoxification enzymes and the susceptibilities of *S. furcifera* to each insecticide (Figure 3). There were positive correlations between the LC_50_ values of nitenpyram, thiamethoxam and clothianidin and the activity of EST. In contrast, no significant correlations were found between the activities of glutathione *S*-transferase and the LC_50_ values of the insecticides, which was also the result for P450 activity.

## 4. Discussion

Chemical insecticides remain the rational and primary tool for controlling pests [16,20,21,22]. Insecticide resistance monitoring is an important task for effective pest control and understanding insect resistance levels and biochemical resistance mechanisms to insecticides is the basis of integrated pest management (IPM) [23]. This study clarified the current status of the resistance of *S. furcifera* to 10 insecticides in six provinces of China from 2019 to 2020, which was of great value for the resistance monitoring and management of the pest.

Neonicotinoid insecticides are the most important chemical insecticides and are widely used to control various sucking pests [24,25]. Previous studies have suggested that *S. furcifera* has developed low to moderate resistance to neonicotinoid insecticides such as imidacloprid, nitenpyram, clothianidin, thiamethoxam and dinotefuran [26,27]. At present, the field population of *S. furcifera* still maintains low to moderate resistance to this type of insecticide, while the resistance level to some neonicotinoid insecticides showed an increasing trend in the period from 2019–2020. For instance, the resistance level of *S. furcifera* to imidacloprid in the AHHF-2020 population (RR = 62.55) nearly tripled compared with AHLA-2019 (RR = 24.27). The dramatic difference in imidacloprid resistance between these two fields suggests that resistance development to a compound can be area specific [7,16]. For other neonicotinoid insecticides, such as thiamethoxam, nitenpyram, dinotefuran and clothianidin, the resistance level of *S. furcifera* also increased slightly. However, the resistance of *S. furcifera* to nitenpyram and thiamethoxam was still maintained at low levels, indicating that it could be used interchangeably with other neonicotinoid insecticides. The above results indicated that neonicotinoid insecticides were still an important measure for farmers to control the whitebacked planthoppers. We recommend using compounds with different modes of action to delay the resistance to neonicotinoid insecticides.

Buprofezin, isoprocarb, ethofenprox and chlorpyrifos have been used to control *S. furcifera* with a long history in most rice-growing areas of southern Vietnam, China, Thailand and Malaysia [28,29,30,31]. Previous studies have suggested that *S. furcifera* has developed a high level of resistance to chlorpyrifos and buprofezin [2,32,33]. Similar to the results of previous studies, our results showed that *S. furcifera* still had a high level of resistance to chlorpyrifos and buprofezin [7]. We speculated that the long-distance migratory behavior of *S. furcifera* may be one of the reasons for this phenomenon. In addition, the resistance ratio of *S. furcifera* to isoprocarb and ethofenprox remained low, which may be due to the low frequency of use of the two insecticides in the monitored areas. Sulfoxaflor is a new type of sulfoximine insecticide that has high control efficacy on rice planthoppers [34,35,36]. Similar to the results in our previous study, *S. furcifera* developed moderate resistance levels to sulfoxaflor [7]. Studies have shown that resistance to sulfoxaflor was significantly correlated with neonicotinoid insecticides in *N. lugens*; therefore, we recommended that sulfoxaflor be used cautiously to control *S. furcifera* [37].

Enzyme activities can play a key role in understanding the patterns of resistance and insect susceptibility to chemicals [38]. Many previous reports found that P450, EST and GST were commonly the primary detoxification enzymes that help insect pests degrade a different type of xenobiotic [39,40,41]. Our results indicated that significant differences existed in the activity of detoxification enzymes between different populations. For instance, the EST activities were significantly different between the populations of JXNC-2019 and HNXY-2019, AHLA-2019, HNNX-2019, HBJM-2019 and HBCB-2019, which suggested that EST may be involved in the resistance of these populations to insecticides. Similarly, the P450 activities of AHHF-2020 and HNNX-2020 were higher than those of HBXZ-2020, HBJL-2020, HBDY-2020, HNXY-2020, JXNC-2020, HBJM-2020, HBWX-2020 and HBCB-2020, while enhancing P450 activity has been confirmed to be an important mechanism for the detoxification and metabolism of pests [42,43,44].

To delay or prevent the evolution of resistance, the most frequently used strategy is to alternate/rotate/mix pesticides [45]. Therefore, the cross-resistance between insecticides is worthy of attention. Previous studies have shown that significant cross-resistance exists between neonicotinoid insecticides in some pests. For instance, our results indicated that resistance to imidacloprid in *S. furcifera* was significantly associated with thiamethoxam, nitenpyram, dinotefuran, clothianidin and sulfoxaflor, which also occurred in small planthoppers, brown planthoppers, potato beetles, cotton aphids and peach aphids [37,46,47,48,49]. In addition, no risk of cross-resistance occurred between ethofenprox and neonicotinoid insecticides; therefore, it could be used as an important alternative insecticide for the control of whitebacked planthoppers. In our study, we found that the activity of EST was significantly correlated with nitenpyram, thiamethoxam clothianidin. Previous studies have shown that EST activities are related to the susceptibility of brown planthoppers to sulfoxaflor and nitenpyram [37,39,49]. Moreover, positively significant correlations were demonstrated between the activity of EST and clothianidin, nitenpyram and triflumezopyrim in field populations of *S. furcifera* [7]. These results suggested that EST might play an important role in the resistance of *S. furcifera* to nitenpyram, dinotefuran, clothianidin and imidacloprid.

## 5. Conclusions

Our findings revealed that field populations of whitebacked planthoppers have developed high levels of resistance to chlorpyrifos and buprofezin; therefore, we suggest that the use of chlorpyrifos and buprofezin should be suspended to control this pest. Additionally, although neonicotinoid insecticides are highly effective, the risk of cross-resistance should be given special attention. We recommend alternating the use of compounds with different modes of action that are more selectively compatible with natural enemies to slow down the resistance development of *S. furcifera.* Overall, this study provided useful data for the controlling of whitebacked planthoppers in the field, including the rational use of chemical insecticide, reducing the risk of control failure and input costs, which is in line with the definition of IPM.

## Figures and Tables

**Table 1:** Information of *S. furcifera* collected from rice paddy fields of China.

Populations	Sampling Sites	Collection Date	Site	Insect Stage	Number
HBCB-2019	Chibi, Hubei	15 July 2019	29°31′ N, 113°42′ E	Nymph and adult	1206
HBWX-2019	Wuxue, Hubei	6 September 2019	29°57′ N, 115°36′ E	Nymph and adult	1374
HBJM-2019	Jingmen, Hubei	5 September 2019	30°32′ N, 112°18′ E	Nymph and adult	1453
HNNX-2019	Ningxiang, Hunan	20 June 2019	28°10′ N, 112°33′ E	Nymph and adult	1069
JXNC-2019	Nanchang, Jiangxi	12 June 2019	28°32′ N, 115°58′ E	Nymph and adult	1146
AHLA-2019	Luan, Anhui	10 July 2019	31°32′ N, 116°18′ E	Nymph and adult	1073
HNXY-2019	Xinyang, Henan	14 July 2019	31°58′ N, 115°24′ E	Nymph and adult	1421
HBDY-2020	Dangyang, Hubei	19 July 2020	30°59′ N, 111°52′ E	Nymph and adult	1306
HBCB-2020	Chibi, Hubei	21 July 2020	29°31′ N, 113°42′ E	Nymph and adult	1154
HBWX-2020	Wuxue, Hubei	8 August 2020	30°07′ N, 115°36′ E	Nymph and adult	1073
HBJL-2020	Jianli, Hubei	25 July 2020	29°49′ N, 112°54′ E	Nymph and adult	1209
HBJM-2020	Jingmen, Hubei	14 July 2020	30°54′ N, 112°14′ E	Nymph and adult	1043
HBXZ-2020	Xinzhou, Hubei	14 July 2020	30°51′ N, 114°37′ E	Nymph and adult	1194
HNNX-2020	Ningxiang, Hunan	3 August 2020	28°10′ N, 112°33′ E	Nymph and adult	1206
JXNC-2020	Nanchang, Jiangxi	25 July 2020	28°32′ N, 115°58′ E	Nymph and adult	1311
AHHF-2020	Luan, Anhui	12 August 2020	31°31′ N, 116°37′ E	Nymph and adult	1248
HNXY-2020	Xinyang, Henan	15 August 2020	31°58′ N, 115°24′ E	Nymph and adult	1076
GZQN-2020	Qiannan, Guizhou	15 August 2020	25°59′ N, 106°35′ E	Nymph and adult	1108

**Table 2:** The susceptibility baseline of *S. furcifera* to insecticides.

Group	Insecticide	LC_50_ (95%CI ^a^) (mg/L)	Reference
neonicotinoids	imidacloprid	0.11 (0.06–0.17)	Su et al., 2013
	thiamethoxam	0.18 (0.11–0.23)	Su et al., 2013
	nitenpyram	0.27 (0.19–0.36)	Zhang et al., 2017
	dinotefuran	0.20 (0.16–0.25)	Li et al., 2020
	clothianidin	0.15 (0.09–0.21)	Zhang et al., 2017
Sulfoximines	sulfoxaflor	0.50 (0.33–0.66)	Li et al., 2020
Insect growth regulators	buprofezin	0.04 (0.03–0.06)	Li et al., 2020
Carbamates	isoprocarb	9.42 (6.97–11.80)	Li et al., 2020
Pyrethroids	ethofenprox	34.61 (20.28–52.76)	Li et al., 2020
Organophosphates	chlorpyrifos	0.24 (0.17–0.31)	Li et al., 2020

^a^ CI, confidence interval.

**Table 3:** Resistance levels of *S. furcifera* field populations to insecticides, including (**A**) imidacloprid and thiamethoxam, (**B**) nitenpyram and dinotefuran, (**C**) clothianidin and sulfoxaflor, (**D**) buprofezin and chlorpyrifos, (**E**) isoprocarb and ethofenprox.

**A**												
**Population**	**Imidacloprid**						**Thiamethoxam**					
	**No.**	**Slope (SE ^a^)**	**LC_50_ (95% CI ^b^; mg/L)**	**χ^2^ (df)**	***p* Value**	**RR ^c^**	**No.**	**Slope (SE ^a^)**	**LC_50_ (95% CI ^b^; mg/L)**	**χ^2^ (df)**	***p* Value**	**RR ^c^**
HBCB-2019	270	2.00 (0.27)	0.72 (0.49–0.94)	0.67 (3)	0.88	6.55	270	1.53 (0.23)	1.31 (0.99–1.90)	5.62 (3)	0.13	7.28
HBWX-2019	270	1.88 (0.25)	0.23 (0.17–0.30)	0.89 (3)	0.83	2.09	270	1.64 (0.23)	0.24 (0.17–0.32)	3.08 (3)	0.38	1.33
HBJM-2019	270	2.32 (0.27)	1.43 (1.14–1.74)	1.11 (3)	0.77	13.00	270	1.67 (0.23)	0.98 (0.77–1.30)	0.69 (3)	0.88	5.44
HNNX-2019	270	1.72 (0.24)	1.63 (1.11–2.14)	0.82 (3)	0.84	14.82	270	2.02 (0.25)	1.51 (1.21–1.87)	4.37 (3)	0.22	8.39
JXNC-2019	270	1.90 (0.28)	2.28 (1.66–3.82)	1.63 (3)	0.65	20.73	270	1.62 (0.25)	2.08 (1.50–3.50)	1.84 (3)	0.61	11.56
AHLA-2019	270	2.48 (0.28)	2.67 (2.19–3.21)	2.16 (3)	0.54	24.27	270	1.70 (0.24)	2.99 (2.29–4.35)	4.08 (3)	0.25	16.61
HNXY-2019	270	1.65 (0.23)	1.46 (1.08–1.88)	2.04 (3)	0.56	13.27	270	1.54 (0.24)	1.96 (1.41–3.29)	1.17 (3)	0.11	10.88
HBDY-2020	270	1.68 (0.23)	1.19 (0.92–1.63)	1.78 (3)	0.58	10.82	270	1.81 (0.24)	1.07 (0.79–1.35)	0.95 (3)	0.81	5.94
HBCB-2020	270	1.30 (0.22)	1.88 (1.37–2.66)	2.28 (3)	0.19	17.09	270	1.30 (0.22)	1.17 (0.86–1.75)	3.61 (3)	0.31	6.50
HBWX-2020	270	0.82 (0.21)	1.40 (0.77–6.04)	3.20 (3)	0.86	12.73	270	1.17 (0.21)	0.42 (0.30–0.61)	2.29 (3)	0.51	2.33
HBJL-2020	270	1.17 (0.21)	2.94 (2.09–4.61)	3.72 (3)	0.84	26.73	270	1.84 (0.26)	1.56 (1.17–2.33)	3.54 (2)	0.17	8.67
HBJM-2020	270	2.33 (0.27)	2.38 (1.97–2.94)	2.45 (3)	0.87	21.64	270	1.86 (0.25)	2.53 (2.00–3.43)	1.31 (3)	0.73	14.06
HBXZ-2020	270	1.32 (0.22)	2.28 (1.68–3.20)	0.79 (3)	0.42	20.73	270	1.27 (0.22)	3.22 (2.29–5.46)	3.73 (3)	0.29	17.89
HNNX-2020	270	1.43 (0.22)	3.25 (2.43–4.82)	2.44 (3)	0.40	29.55	270	2.08 (0.25)	1.50 (1.21–1.85)	3.06 (3)	0.38	8.33
JXNC-2020	270	2.13 (0.25)	2.02 (1.64–2.49)	0.57 (3)	0.61	18.36	270	2.10 (0.25)	1.79 (1.45–2.22)	3.09 (3)	0.38	9.94
AHHF-2020	270	1.58 (0.26)	6.88 (4.64–13.67)	2.18 (3)	0.80	62.55	270	2.20 (0.26)	2.13 (1.74–2.69)	0.31 (3)	0.96	11.83
HNXY-2020	270	2.58 (0.31)	2.21 (1.68–2.71)	1.59 (3)	0.29	20.09	270	1.84 (0.26)	0.90 (0.60–1.19)	2.09 (3)	0.55	5.00
GZQN-2020	270	2.35 (0.29)	3.86 (3.09–5.24)	3.83 (3)	0.21	35.09	270	1.70 (0.28)	3.01 (2.01–6.19)	1.88 (3)	0.60	16.72
**B**												
**Population**	**Nitenpyram**						**Dinotefuran**					
	**No.**	**Slope (SE ^a^)**	**LC_50_ (95% CI ^b^; mg/L)**	**χ^2^ (df)**	***p* Value**	**RR ^c^**	**No.**	**Slope (SE ^a^)**	**LC_50_ (95% CI ^b^; mg/L)**	**χ^2^ (df)**	***p* Value**	**RR ^c^**
HBCB-2019	270	1.56 (0.23)	0.67 (0.51–0.96)	3.34 (3)	0.34	2.48	270	2.12 (0.25)	1.92 (1.55–2.36)	0.57 (3)	0.90	9.60
HBWX-2019	270	1.88 (0.24)	0.27 (0.20–0.34)	3.51 (3)	0.32	1.00	270	2.25 (0.27)	0.27 (0.21–0.33)	1.31 (3)	0.73	1.35
HBJM-2019	270	1.56 (0.26)	0.12 (0.06–0.17)	2.47 (3)	0.48	0.67	270	2.14 (0.28)	1.00 (0.71–1.27)	0.32 (3)	0.96	5.00
HNNX-2019	270	2.79 (0.30)	1.44 (1.20–1.70)	2.02 (3)	0.57	5.33	270	2.76 (0.31)	2.70 (2.27–3.30)	0.31 (3)	0.96	13.50
JXNC-2019	270	2.35 (0.27)	1.05 (0.87–1.32)	0.04 (3)	0.99	3.89	270	1.69 (0.25)	4.75 (3.50–7.56)	1.14 (3)	0.77	23.75
AHLA-2019	270	1.85 (0.24)	1.79 (1.42–2.28)	2.00 (3)	0.57	6.63	270	2.03 (0.25)	2.13 (1.62–2.65)	1.05 (3)	0.79	10.65
HNXY-2019	270	1.70 (0.24)	2.61 (2.02–3.64)	4.10 (3)	0.25	9.67	270	2.72 (0.37)	1.28 (0.87–1.65)	1.59 (3)	0.66	6.40
HBDY-2020	270	2.00 (0.33)	0.41 (0.20–0.61)	1.78 (3)	0.62	1.52	270	2.10 (0.25)	1.93 (1.56–2.38)	3.33 (3)	0.34	9.65
HBCB-2020	270	2.03 (0.25)	1.08 (0.83–1.35)	2.28 (3)	0.52	4.00	270	2.12 (0.28)	1.76 (1.20–2.29)	5.24 (3)	0.16	8.80
HBWX-2020	270	2.75 (0.31)	0.59 (0.49–0.74)	3.20 (3)	0.36	2.19	270	1.58 (0.25)	1.16 (0.81–2.09)	2.42 (3)	0.49	5.80
HBJL-2020	270	1.73 (0.24)	1.48 (1.14–2.13)	3.72 (3)	0.29	5.48	270	2.06 (0.26)	6.30 (5.06–8.32)	3.82 (3)	0.28	31.50
HBJM-2020	270	1.91 (0.24)	1.44 (1.13–1.80)	2.45 (3)	0.48	5.33	270	1.77 (0.24)	3.25 (2.54–4.49)	0.62 (3)	0.89	16.25
HBXZ-2020	270	1.73 (0.23)	2.25 (1.76–3.02)	0.79 (3)	0.85	8.33	270	2.61 (0.32)	3.72 (3.02–4.92)	2.99 (3)	0.39	18.60
HNNX-2020	270	2.16 (0.25)	1.67 (1.36–2.06)	2.44 (3)	0.49	6.19	270	1.44 (0.23)	3.52 (2.62–5.33)	2.10 (3)	0.55	17.60
JXNC-2020	270	2.03 (0.26)	0.98 (0.73–1.23)	0.57 (3)	0.90	3.63	270	1.62 (0.22)	2.10 (1.62–2.74)	1.18 (3)	0.76	10.50
AHHF-2020	270	2.34 (0.27)	1.57 (1.29–1.91)	2.18 (3)	0.53	5.81	270	1.57 (0.24)	3.28 (2.48–4.78)	0.90 (3)	0.83	16.40
HNXY-2020	270	2.02 (0.25)	1.14 (0.88–1.42)	1.59 (3)	0.67	4.22	270	1.77 (0.25)	2.12 (1.47–2.76)	2.56 (3)	0.47	10.60
GZQN-2020	270	2.33 (0.27)	1.02 (0.84–1.27)	3.83 (3)	0.28	3.78	270	2.15 (0.30)	10.29 (7.76–15.90)	1.80 (3)	0.62	51.45
**C**												
**Population**	**Clothianidin**						**Sulfoxaflor**					
	**No.**	**Slope (SE ^a^)**	**LC_50_ (95% CI ^b^; mg/L)**	**χ^2^ (df)**	***p* Value**	**RR ^c^**	**No.**	**Slope (SE ^a^)**	**LC_50_ (95% CI ^b^; mg/L)**	**χ^2^ (df)**	***p* Value**	**RR ^c^**
HBCB-2019	270	2.10 (0.26)	1.17 (0.92–1.45)	0.91 (3)	0.82	7.80	270	2.49 (0.32)	3.31 (2.32–4.22)	1.36 (3)	0.82	6.62
HBWX-2019	270	2.42 (0.28)	0.27 (0.21–0.33)	0.63 (3)	0.89	1.80	270	2.31 (0.28)	2.47 (1.90–3.03)	3.10 (3)	0.38	4.94
HBJM-2019	270	2.64 (0.32)	0.83 (0.62–1.03)	1.80 (3)	0.61	5.53	270	2.86 (0.32)	2.86(2.33–3.39)	2.50 (3)	0.48	5.72
HNNX-2019	270	2.32 (0.27)	1.26 (1.01–1.52)	0.99 (3)	0.80	8.40	270	2.22 (0.26)	8.08 (6.61–10.21)	0.73 (3)	0.86	16.16
JXNC-2019	270	1.65 (0.26)	2.53 (1.75–4.69)	1.76 (3)	0.62	16.87	270	1.73 (0.23)	7.51 (5.91–9.89)	8.31 (3)	0.04	15.02
AHLA-2019	270	1.66 (0.23)	1.49 (1.15–1.92)	2.39 (3)	0.50	9.93	270	1.88 (0.24)	8.14 (6.50–10.54)	0.67 (3)	0.88	16.28
HNXY-2019	270	1.52 (0.24)	1.72 (1.26–2.75)	5.95 (3)	0.11	11.47	270	2.51 (0.29)	2.81 (2.33–3.50)	0.21 (3)	0.98	5.62
HBDY-2020	270	2.06 (0.27)	0.77 (0.54–0.99)	3.70 (3)	0.30	5.13	270	3.28 (0.23)	3.46 (2.45–4.33)	1.45 (3)	0.70	6.92
HBCB-2020	270	2.25 (0.27)	1.05 (0.82–1.29)	4.99 (3)	0.17	7.00	270	1.89 (0.24)	3.17 (2.46–3.96)	2.19 (3)	0.53	6.34
HBWX-2020	270	2.53 (0.28)	0.44 (0.37–0.54)	4.47 (3)	0.22	2.93	270	1.55 (0.23)	7.08 (5.34–10.45)	1.32 (3)	0.73	14.16
HBJL-2020	270	1.69 (0.25)	1.89 (1.40–3.01)	2.92 (3)	0.40	12.60	270	1.88 (0.25)	7.01 (5.50–9.71)	3.16 (3)	0.37	14.02
HBJM-2020	270	2.51 (0.28)	1.29 (1.06–1.55)	1.90 (3)	0.59	8.60	270	4.08 (0.46)	6.39 (5.44–7.34)	3.09 (3)	0.38	12.78
HBXZ-2020	270	1.96 (0.25)	2.28 (1.83–2.99)	3.46 (3)	0.33	15.20	270	2.09 (0.25)	8.44 (6.84–10.47)	2.36 (3)	0.50	16.88
HNNX-2020	270	2.02 (0.25)	1.19 (0.92–1.47)	2.37 (3)	0.50	7.93	270	2.32 (0.27)	5.92 (4.72–7.19)	2.83 (3)	0.42	11.84
JXNC-2020	270	2.12 (0.25)	1.97 (1.60–2.47)	2.17 (3)	0.54	13.13	270	1.81 (0.24)	6.52 (5.02–8.22)	1.72 (3)	0.63	13.04
AHHF-2020	270	1.94 (0.25)	2.07 (1.66–2.66)	1.65 (3)	0.65	13.80	270	2.25 (0.27)	5.79 (4.57–7.06)	2.03 (3)	0.57	11.58
HNXY-2020	270	1.74 (0.23)	1.79 (1.38–2.28)	2.64 (3)	0.45	11.93	270	2.47 (0.29)	2.35 (1.80–2.87)	3.13 (3)	0.37	4.70
GZQN-2020	270	1.39 (0.24)	2.60 (1.73–5.35)	1.63 (3)	0.65	17.33	270	1.77 (0.25)	16.13 (12.30–23.74)	0.77 (3)	0.86	32.26
**D**												
**Population**	**Buprofezin**						**Chlorpyrifos**					
	**No.**	**Slope (SE ^a^)**	**LC_50_ (95% CI ^b^; mg/L)**	**χ^2^ (df)**	***p* Value**	**RR ^c^**	**No.**	**Slope (SE ^a^)**	**LC_50_ (95% CI ^b^; mg/L)**	**χ^2^ (df)**	***p* Value**	**RR ^c^**
HBCB-2019	270	1.89 (0.24)	3.90 (3.09–4.90)	0.86 (3)	0.84	97.50	270	2.44 (0.28)	30.72 (25.28–39.14)	1.80 (3)	0.83	128.00
HBWX-2019	270	1.72 (0.23)	3.71 (2.87–4.74)	4.39 (3)	0.22	92.75	270	1.74 (0.23)	20.40 (15.96–26.16)	3.27 (3)	0.35	85.00
HBJM-2019	270	1.93 (0.24)	3.42 (2.69–4.26)	1.01 (3)	0.80	85.50	270	3.09 (0.33)	20.67 (17.60–24.35)	7.13 (3)	0.07	86.13
HNNX-2019	270	1.95 (0.26)	3.25 (2.33–4.14)	1.01 (3)	0.80	81.25	270	3.57 (0.40)	54.07 (46.52–64.75)	5.00 (3)	0.17	225.29
JXNC-2019	270	1.43 (0.23)	13.26 (9.55–21.97)	4.70 (3)	0.20	331.50	270	2.39 (0.27)	32.51 (26.36–39.23)	4.72 (3)	0.19	135.46
AHLA-2019	270	1.57 (0.23)	6.80 (5.22–9.03)	1.19 (3)	0.75	170.00	270	2.02 (0.26)	73.00 (57.61–100.50)	0.81 (3)	0.85	304.17
HNXY-2019	270	1.99 (0.25)	5.01 (4.04–6.39)	6.49 (3)	0.09	125.25	270	1.96 (0.24)	46.58 (37.47–59.03)	6.34 (3)	0.10	194.08
HBDY-2020	270	1.89 (0.24)	4.64 (3.58–5.80)	1.71 (3)	0.64	105.45	270	2.92 (0.32)	39.04 (32.97–46.13)	0.38 (3)	0.94	162.67
HBCB-2020	270	1.65 (0.23)	5.20 (4.05–6.96)	1.30 (3)	0.73	118.18	270	2.68 (0.29)	15.33 (12.81–18.27)	2.37 (3)	0.50	63.88
HBWX-2020	270	1.48 (0.22)	5.70 (4.32–8.04)	2.25 (3)	0.52	129.55	270	2.14 (0.27)	11.46 (8.53–14.32)	1.50 (3)	0.68	47.75
HBJL-2020	270	2.33 (0.28)	7.13 (5.75–9.48)	1.45 (3)	0.69	162.05	270	3.07 (0.34)	26.34 (21.29–31.22)	2.14 (3)	0.54	109.75
HBJM-2020	270	1.70 (0.23)	5.05 (3.96–6.68)	3.74 (3)	0.29	114.77	270	3.00 (0.32)	21.34 (18.14–25.25)	4.24 (3)	0.24	88.92
HBXZ-2020	270	1.45 (0.23)	6.60 (4.94–9.77)	0.34 (3)	0.95	150.00	270	2.36 (0.31)	43.69 (34.31–61.90)	1.74 (3)	0.63	182.04
HNNX-2020	270	1.57 (0.23)	4.43 (3.23–5.76)	3.57 (3)	0.31	100.68	270	3.20 (0.34)	39.65 (33.81–46.46)	2.47 (3)	0.48	165.21
JXNC-2020	270	1.59 (0.23)	5.51 (4.18–7.16)	0.69 (3)	0.87	137.75	270	2.06 (0.25)	22.88 (18.55–28.69)	1.34 (3)	0.72	95.33
AHHF-2020	270	1.58 (0.23)	3.88 (2.76–5.07)	2.49 (3)	0.48	88.18	270	2.27 (0.26)	45.34 (37.32–55.83)	0.30 (3)	0.96	188.92
HNXY-2020	270	1.51 (0.22)	4.30 (3.27–5.73)	1.37 (3)	0.71	97.73	270	1.68 (0.24)	39.41 (29.88–58.46)	1.49 (3)	0.69	164.21
GZQN-2020	270	1.98 (0.27)	8.82 (6.76–13.01)	3.18 (3)	0.36	200.45	270	2.45 (0.28)	18.54 (15.289–22.31)	1.46 (3)	0.69	77.25
**E**												
**Population**	**Isoprocarb**						**Ethofenprox**					
	**No.**	**Slope (SE ^a^)**	**LC_50_ (95% CI ^b^; mg/L)**	**χ^2^ (df)**	***p* Value**	**RR ^c^**	**No.**	**Slope (SE ^a^)**	**LC_50_ (95% CI ^b^; mg/L)**	**χ^2^ (df)**	***p* Value**	**RR ^c^**
HBCB-2019	270	2.63 (0.29)	97.84 (81.69–116.95)	0.88 (3)	0.83	10.39	270	1.25 (0.22)	435.50 (304.77–770.96)	0.53 (3)	0.91	12.58
HBWX-2019	270	2.16 (0.26)	91.57 (73.98–112.21)	2.52 (3)	0.47	9.72	270	1.86 (0.24)	252.99 (201.67–327.75)	5.73 (3)	0.13	7.31
HBJM-2019	270	2.53 (0.28)	124.26 (103.72–152.18)	2.79 (3)	0.43	13.20	270	2.13 (0.26)	327.97(263.69–434.08)	1.99 (3)	0.57	9.47
HNNX-2019	270	2.64 (0.30)	86.96 (73.33–104.62)	4.89 (3)	0.18	9.23	270	1.62 (0.25)	575.55 (420.53–933.75)	1.91 (3)	0.59	16.63
JXNC-2019	270	1.43 (0.24)	215.22 (150.02–390.68)	0.04 (3)	0.99	22.85	270	1.38(0.22)	209.86 (154.59–322.82)	2.46 (3)	0.48	6.06
AHLA-2019	270	1.65 (0.23)	75.78 (58.24–97.76)	1.52 (3)	0.68	8.04	270	1.61 (0.24)	275.60 (202.47–440.60)	0.53 (3)	0.91	7.96
HNXY-2019	270	1.88 (0.24)	26.14 (20.86–33.95)	4.80 (3)	0.19	2.78	270	2.14 (0.26)	256.60 (209.20–324.06)	0.63 (3)	0.89	7.41
HBDY-2020	270	1.70 (0.24)	134.86 (104.16–190.17)	2.07 (3)	0.56	14.32	270	1.27 (0.22)	301.41 (219.77–458.33)	0.73 (3)	0.87	8.71
HBCB-2020	270	2.23 (0.26)	68.42 (55.22–83.31)	0.64 (3)	0.89	7.26	270	2.26 (0.26)	232.67 (191.46–287.45)	2.46 (3)	0.48	6.72
HBWX-2020	270	2.32 (0.27)	116.69 (96.33–143.72)	2.00 (3)	0.57	12.39	270	1.33 (0.22)	311.79 (229.35–470.59)	2.85 (3)	0.41	9.01
HBJL-2020	270	2.04 (0.25)	140.85 (113.65–182.22)	2.57 (3)	0.46	14.95	270	1.60 (0.26)	679.15 (459.97–1333.14)	3.75 (3)	0.29	19.62
HBJM-2020	270	2.61 (0.29)	101.95 (85.21–122.22)	6.32 (3)	0.10	10.82	270	2.08 (0.26)	303.41 (244.62–396.48)	4.52 (3)	0.21	8.77
HBXZ-2020	270	2.25 (0.28)	171.83 (138.58–227.68)	6.35 (3)	0.10	18.24	270	1.17 (0.21)	268.36 (191.17–410.57)	5.27 (3)	0.15	7.75
HNNX-2020	270	1.89 (0.25)	120.27 (95.57–160.13)	1.06 (3)	0.79	12.77	270	1.10 (0.22)	561.88 (362.20–1273.63)	0.51 (3)	0.92	16.23
JXNC-2020	270	1.97 (0.25)	101.17 (81.44–129.51)	3.98 (3)	0.26	10.74	270	1.54 (0.23)	232.41 (173.47–353.93)	2.45 (3)	0.49	6.72
AHHF-2020	270	2.13 (0.26)	60.14 (47.36–73.85)	1.34 (3)	0.72	6.38	270	1.34 (0.22)	226.23 (167.47–330.85)	0.64 (3)	0.89	6.54
HNXY-2020	270	2.00 (0.26)	79.05 (61.69–111.69)	4.31 (3)	0.23	8.39	270	1.59 (0.23)	304.42 (233.94–426.32)	3.59 (3)	0.31	8.80
GZQN-2020	270	1.98 (0.26)	183.31 (144.14–253.85)	1.42 (3)	0.70	19.46	270	1.71 (0.27)	665.53 (458.41–1255.03)	2.62 (3)	0.45	19.23

^a^ SE, standard error; ^b^ 95% CI, confidence interval; df, degrees of freedom; ^c^ RR, resistance ratio.

## Data Availability

Not applicable.

## Supplementary Materials

The following are available online at https://www.mdpi.com/article/10.3390/insects12121078/s1, Table S1: Concentrations of immersion for each insecticide.
