# Supplementary material for: Insecticide Resistance Monitoring in Field Populations of the Whitebacked Planthopper Sogatella furcifera (Horvath) in China, 2019–2020
Source: Insects. 2021 Nov 30;12(12):1078. doi: 10.3390/insects12121078 (PMC8706372; doi:10.3390/insects12121078)
Supplement: Supplementary file 1 [file insects-12-01078-s001.zip › insects-1395246-supplementary.pdf]

Table S1 Concentrations of immersion for each insecticide

Insecticide	Concentration- 2019 (mg/L)	Concentration- 2020 (mg/L)
Imidacloprid	0/0.4/0.8/1.6/3.2/6.4	0/0.4/0.8/1.6/3.2/6.4
Thiamethoxam	0/0.2/0.4/0.8/1.6/3.2	0/0.2/0.4/0.8/1.6/3.2
Nitenpyram	0/0.1/0.4/0.8/1.6/3.2	0/0.4/0.8/1.6/3.2/6.4
Dinotefuran	0/1/2/4/8/16	0/1/2/4/8/16
Clothianidin	0/0.2/0.4/0.8/1.6/3.2	0/0.4/0.8/1.6/3.2/6.4
Sulfoxaflor	0/1/2/4/8/16	0/1/2/4/8/16
Buprofezin	0/1/2/4/8/16	0/1/2/4/8/16
Isoprocarb	0/100/200/400/800/1200	0/20/40/80/160/320
Ethofenprox	0/100/200/400/800/1200	0/50/100/200/400/800
Chlorpyrifos	0/10/20/40/80/160	0/4/8/16/32/64
